# IRGL-RRI: interpretable graph representation learning for plant RNA–RNA interaction discovery

**DOI:** 10.3389/fpls.2025.1617495

**Published:** 2025-06-05

**Authors:** Qingquan Liao, Xuchong Liu, Wei Zhao, Yu Tong, Fangzheng Xu, Xinxin Liu, Yifan Chen

**Affiliations:** ^1^ Department of Information Technology, Hunan Police Academy, Changsha, China; ^2^ School of Data Science and Artificial Intelligence, Wenzhou University of Technology, Wenzhou, China; ^3^ Institute of Artificial Intelligence Application, College of Computer and Mathematics, Central South University of Forestry and Technology, Changsha, China

**Keywords:** plant RNA-RNA interactions, plant gene functions, graph representation learning, interpretability, regularization

## Abstract

Plant RNAs are crucial for plant gene expression and protein synthesis. They modulate the spatial structure of themselves and associated molecules, thereby influencing transcription, translation and gene expression regulation. Molecular biology experiments enhance our understanding of plant RNA-RNA interactions (RRIs), yet their complex structure and dynamic properties render these experiments expensive and time-consuming. Recent advances in deep learning have transformed plant RNA research and improved RRI prediction efficiency. However, these methods still struggle with poor prediction accuracy. To address this, this study proposes an interpretable graph representation model for accurate plant RRI prediction. The model enriches sample information by extracting features of different bases from plant RNA data and reconstructs these features using an algorithmic hierarchy approach to capture more complex patterns. A graph representation based on a masking strategy and regularization enhances RNA feature extraction. Furthermore, an RRI modeling approach combining Kolmogorov-Arnold Networks (KAN) and multi-scale fusion is proposed to deeply resolve the complex dynamic interaction mechanisms of RRIs and improve model interpretability. Performance evaluations and case studies on publicly available datasets demonstrate that the proposed model can accurately identify potential RRIs, indicating its potential as a powerful tool for plant gene function annotation. Our data and code are available at: https://github.com/Lqingquan/IGRL-RRI.

## Introduction

Plant RNAs are essential for genetic information transfer and protein synthesis in organisms. They modulate the high-level structure of themselves and their interacting molecules and fine-tune transcription, translation, and gene expression regulatory networks. An increasing number of studies have demonstrated that different plant RNAs can interact to form regulatory networks and participate in numerous life activities (Chen et al., 2019; Cao et al., 2021; Wang et al., 2022). Plant long non-coding RNAs (lncRNAs) can bind to mRNAs via base complementary pairing, regulating their stability or translation efficiency. For instance, some lncRNAs prolong mRNA half-life by forming double-stranded structures (Cao et al., 2021). In plants, lncRNAs also play a key role in stress response. For example, the COOLAIR lncRNA affects flowering time in *Arabidopsis thaliana* by regulating the expression of the FLOWERING LOCUS C (FLC) gene to adapt to different environmental conditions. Circular RNAs (circRNAs), on the other hand, can counteract the inhibitory effect of microRNAs (miRNAs) on the mRNAs of target genes by binding miRNA molecules. For example, circRNA_CDR1 can be involved in the regulation of neurodegenerative diseases by binding miRNA-7 and promoting the expression of its target genes (Xue, 2022). In rice (*Oryza sativa*), certain circRNAs can act as competing endogenous RNAs (ceRNAs) for miRNAs, regulating gene expression and thereby affecting plant tolerance to abiotic stresses such as salt stress and drought.

Plant RRIs orchestrate gene expression networks through three key mechanisms: chromatin structure regulation, post-transcriptional modification, and signaling pathway modulation. In rice, chromatin-bound RNAs establish R-loops via long-range interactions, regulating 55% of cross-chromosomal gene interactions (Xiao et al., 2022). In plant immunity, conserved trans-lncRNA pairs in tea plants enhance fungal pathogen resistance through jasmonic acid pathway suppression, with observed conservation across multiple crops (Sun et al., 2024). Furthermore, m6A modifications balance defense-gene expression and growth regulation, enabling optimized plant responses to biotic stressors while demonstrating RNA epigenetic editing’s agricultural potential (Ge et al., 2025). Collectively, these findings elucidate RRI molecular mechanisms while identifying actionable targets for developing stress-resilient crops. Although plant RNA-RNA interactions (RRIs) are crucial for gene regulation, their experimental validation is often expensive and time-consuming. Moreover, traditional bioinformatics methods have limitations when dealing with complex structures and large-scale data. However, the development of high-throughput sequencing technology and the resulting accumulation of RRI data have made computational prediction feasible. Modern computational methods, especially machine learning and deep learning, are becoming important tools for understanding RRI mechanisms. The main methods for inferring potential RRIs include experimental parsing techniques, a combination of experimental and high-throughput sequencing techniques, and computational methods such as machine learning and deep learning.

Experimental techniques can uncover the structural and functional properties of RNA interactions by directly measuring physical evidence of plant RRIs. Current experimental methods mainly rely on advanced techniques like RIC-seq, which captures RNA spatial interactions in living cells through RNA-binding protein (RBP)-mediated neighbor-joining. For example, Cao et al. used RIC-seq to resolve enhancer RNA (eRNA)-promoter RNA interactions in plants. This revealed how chromatin conformation regulates gene transcription and showed that long non-coding RNAs like CCAT1-5L regulate key gene expression through RNA complex formation (Cao et al., 2021). However, RIC-seq mainly depends on RNA-binding proteins. It cannot directly resolve RNA structure stability and dynamic changes. To better reveal RRI structures, cryo-EM combined with molecular dynamics simulation has become a key tool. Wu et al. used cryo-EM to analyze the 3D structure of chloroplast RNA polymerase. They found it comprises 20 protein subunits and plays a key role in photosynthesis gene transcription (Wu X-X. et al., 2024). Zhang et al. determined the cryo-EM structure of Arabidopsis RNA polymerase V (Pol V), revealing its transcription elongation complex features. Pol V functions in the RNA-mediated DNA methylation pathway by binding to KTF1 and recruiting the Argonaute4/6-siRNA complex. Its active center differs from Pol II, resulting in lower transcriptional activity. But its unique structure allows stable chromatin binding, promoting DNA methylation (Zhang et al., 2023). Crosslinking and neighbor-joining techniques are also widely used in studying spliceosome assembly and transcriptional regulation. The TREX (Targeted RNase H-mediated Extraction) technique has been used to study snRNA-pre-mRNA interactions in plant splicing and reveal intron splicing mechanisms. In Arabidopsis thaliana, crosslinking techniques (e.g., ChIP-seq) combined with Hi-C technology showed that RNA polymerase V and siRNA-Argonaute complexes regulate DNA methylation through chromatin loop formation. This affects plant stress tolerance (Zhang et al., 2023).

Combinatorial experiments and high-throughput sequencing technologies can accurately capture RRIs and their higher-order structures in living cells. These technologies integrate chemical labeling, cross-linking techniques, and high-throughput sequencing. Wu et al. used N3-ketoaldehyde labeling and multifunctional chemical cross-linking agents to capture RRIs and higher-order RNA structures in living cells. This method does not rely on RBPs for local binding. By crosslinking labeled RNA molecules and using high-throughput sequencing, this approach generated single-base resolution RNA interaction profiles (Wu T. et al., 2024). It is suitable for RRI studies in higher organisms and has important applications in microbiology. Chao et al. immobilized RNA-interacting complexes using chemical cross-linking and mapped global RRIs in *Salmonella typhi* and *Klebsiella pneumoniae*. They combined neighboring junctions with deep sequencing to systematically construct RNA-RNA networks in microorganisms. This revealed key RNA regulatory centers and their roles in metabolism and pathogenicity, making it particularly applicable to the functional study of prokaryotic non-coding small RNAs (Westermann et al., 2016). In addition to capturing RRIs using chemical crosslinking and high-throughput sequencing, incorporating RNA-protein complexes can provide more comprehensive interaction information. CLASH (Crosslinking, Ligation, and Sequencing of Hybrids) immobilizes RNA-protein complexes through chemical crosslinking. It uses neighbor-joining technology to connect spatially neighboring RNA molecules (e.g., miRNAs and target mRNAs) into chimeric fragments. High-throughput sequencing then directly resolves the RNA-RNA pairs. Zhang et al. developed the CoPRA model, a deep-learning-based tool for predicting protein-RNA binding affinity. Its primary goal is to address limitations of traditional methods in predicting protein-RNA interactions. For the first time, the model combines a dual-scope pretraining strategy with a multimodal fusion architecture, which significantly enhances prediction accuracy (Han et al., 2025). Improving the joining efficiency of spatially neighboring RNA molecules is crucial for accurately constructing interworking networks during RRI resolution. Douglas M. Anderson’s team developed the Stitch RNA system, a nuclease-mediated mRNA trans-joining technology. Centered on ribozyme-mediated RNA splicing reactions, this system enables traceless trans-joining of segmented mRNA fragments in living cells, generating intact functional mRNAs that translate into large proteins. In the Dysferlin-KO mouse model, injecting the Dysferlin gene delivered by the Stitch RNA system significantly restored protein expression in the quadriceps muscle and other body parts, improving muscle function (Lindley et al., 2024).

The rise of deep learning has made computational prediction methods crucial for analyzing RRIs. For instance, preMLI model uses the Transformer architecture to pre-train on large RNA sequence datasets, learning universal sequence representations. It also employs deep feature mining to fine-tune on plant-specific data, enhancing cross-species prediction accuracy. In miRNA-lncRNA interaction predictions between Arabidopsis and rice, this method improved AUC values by over 10% compared to existing methods (Gao et al., 2021). However, relying solely on sequence information may not fully capture the complex interactions of RNA molecules in biological networks. To address this limitation, computational methods incorporating network topology information, such as graph convolutional networks (GCNs), have become significant. GCNs can integrate multimodal features and construct heterogeneous plant RNA networks. These include sequence similarity, co-expression networks, and functional annotation information. By combining GCNs with a random walk algorithm, node-embedded features can be learned to predict RRI relationships. Yu et al. integrated three computational methods (WGCNA, GGM, BC3NET) to predict functional roles of protein-coding genes and non-coding RNAs including lncRNAs and circRNAs in rice. By analyzing 348 RNA-seq samples, the team constructed a co-expression network revealing regulatory mechanisms underlying key biological processes: floral development, cell wall metabolism, and stress response pathways (Yu et al., 2017). Despite the power of GCNs in modeling RRI networks, RRIs are inherently dynamic and influenced by developmental processes, environmental changes, and stress factors. Generative Adversarial Networks (GANs) have emerged as a promising tool for predicting RRIs. They simulate the dynamics of RRIs under different physiological conditions through adversarial learning between the Generator and Discriminator. When combined with Attention Mechanisms, GANs can capture critical RRI features within regulatory time windows. For example, in studying plant responses to environmental stress, GANs can predict how RRIs dynamically remodel under varying stress conditions. This method successfully predicted the dynamic interaction network between miRNAs and target mRNAs in maize under drought stress. Wang et al. developed RPI-CapsuleGAN, integrating GANs and capsule networks with a convolutional attention module to enhance feature interpretability in biomolecular interaction prediction. The model demonstrated superior RNA-protein interaction performance while resolving tensions between biological data scarcity and model stability through adversarial training. Although designed for RNA-protein systems, its framework is adaptable to plant RNA-RNA interaction studies where dynamic networks (e.g., miRNA-lncRNA-circRNA crosstalk during drought/salt stress) challenge conventional static models in capturing temporal regulatory patterns (Wang et al., 2023).

While GNNs demonstrate potential for plant RRI prediction, three key challenges persist. First, inherent topological sparsity (e.g., low node degrees) and experimental noise compromise message propagation in conventional GNNs, yielding suboptimal RNA representations. Second, feature distribution shifts and structural heterogeneity across species severely degrade generalization in single-species-trained models. Third, while random masking-based augmentation enhances topological robustness, it risks erasing critical interaction patterns.

Currently, deep learning techniques like graph neural networks (GNNs) perform well in plant RRI prediction tasks but still face many challenges. Pre-trained models mainly rely on sequence information, making it difficult to fully capture the structural and dynamic features of RRIs. The collected RNA-RNA data may also contain noise, which can affect model performance. Furthermore, plant RRI datasets typically exhibit three key characteristics: high structural diversity, incomplete information, and substantial cross-species variability. Traditional single-scale approaches struggle to capture complex dependencies effectively. Therefore, this study proposes an interpretable graph representation model to better uncover unknown RRIs. We enrich the sample information by extracting multidimensional features from RNA sequences. These features are then reconstructed using an algorithmic approach to capture more complex patterns. Next, a portion of the RNA-RNA graph is randomly masked according to the Bernoulli distribution and input into a GNN encoder. This step reduces the noise effect and enhances the model’s self-supervised learning capability. We also apply L2 regularization to optimize graph representation learning and minimize the impact of node density imbalance in the RNA-RNA graph on message propagation, thereby improving RNA representation quality. Finally, multi-scale fusion framework synergistically integrates local base-pairing features with global topological patterns, significantly improving robustness against information loss. Additionally, a degree decoder incorporating KAN is introduced. This decoder predicts differences between pre- and post-RNA-RNA maps by modeling deep nonlinear mappings, thus enhancing the model’s adaptability and interpretability. In summary, our contributions are as follows:

(1) We propose an interpretable graph representation model that accurately identifies unknown RRIs with compelling results.
(2) We enhance initial RNA representation by extracting base-level multidimensional features, integrating multiple algorithmic reconstructions to uncover higher-order feature associations.
(3) We design a graph representation method that combines a Bernoulli masking strategy with L2 regularization to improve noise immunity, optimize message propagation, and enhance RNA representation.
(4) We propose an RRI modeling method based on KAN and multi-scale fusion to deeply analyze the complex dynamic interaction mechanisms of RRIs while enhancing model interpretability.

## Materials and methods

### Materials

Non-coding RNAs (ncRNAs) play crucial roles in post-transcriptional regulation. miRNAs and lncRNAs dynamically interact to regulate key biological processes such as gene silencing, cellular differentiation, and stress response through sequence complementarity or protein-mediated mechanisms (Chen et al., 2019; Zhou et al., 2020). This study construct a cross-species plant ncRNA interactions based on a previous experimental framework (Kang et al., 2020). This incorporates interaction data from model organisms (e.g., *Arabidopsis thaliana*, *Oryza sativa*) and economically important crops (e.g., *Glycine max*, *Zea mays*), as detailed in **Table 1**. Additionally, we use sequence pattern features and secondary structure topology features, similar to those in prior work (Kang et al., 2022), as the initial RNA representations in this study.

**Table 1:** Plant species and sample information in this study.

Species	Type	Family	Number of positive samples	Number of negative samloes
*A. thaliana*	Dicotyledon	*Cruciferae*	2500	2500
*G. max*	Dicotyledon	*Leguminosae*	2500	2500
*O. sativa*	Monocotyledon	*Gramineae*	2500	2500
*Z. mays*	Monocotyledon	*Gramineae*	2500	2500

### Methods

RRIs are central to gene expression regulation and are involved in key biological processes such as post-transcriptional modification, splicing regulation, translational repression, and chromatin remodeling. Non-coding RNAs (e.g., lncRNAs, circRNAs) form dynamic interaction networks with other RNA molecules to regulate complex functions like cell fate determination, disease genesis, and viral replication. However, traditional experimental methods (e.g., crosslinked immunoprecipitation sequencing, dual luciferase reporter assays), while able to validate specific RRIs, are low-throughput, reliant on prior assumptions, and struggle to resolve genome-wide RRI networks. Consequently, developing efficient computational methods to discover unknown RRIs is crucial, as existing computational models face challenges in prediction accuracy.

This study proposes an interpretable graph representation model designed to precisely uncover unknown RRIs. Base-level features are extracted from RNA sequences to capture multi-dimensional information, including sequence and structural details. This enriches the input representation and enhances the model’s ability to detect potential interactions. A hierarchical feature reconstruction mechanism is introduced to reorganize the feature space, modeling higher-order and more complex feature interactions to boost representation capabilities. During model training, some RNA-RNA graphs are randomly masked using a Bernoulli distribution and fed into a GNN encoder to improve noise resistance and enhance self-supervised learning. L2 regularization optimizes graph representation learning and reduces the impact of node density imbalances on message propagation, ensuring high-quality RNA representations. A cooperative encoder integrates the outputs of each GNN layer to mitigate message loss from random masking. Additionally, a degree decoder incorporating KAN is introduced. This decoder predicts differences between pre-and post-RNA-RNA graph through deep nonlinear mapping modeling, thereby enhancing the model’s adaptability and interpretability. The following sections detail these principles and techniques.

#### Model overview

**Figure 1:**
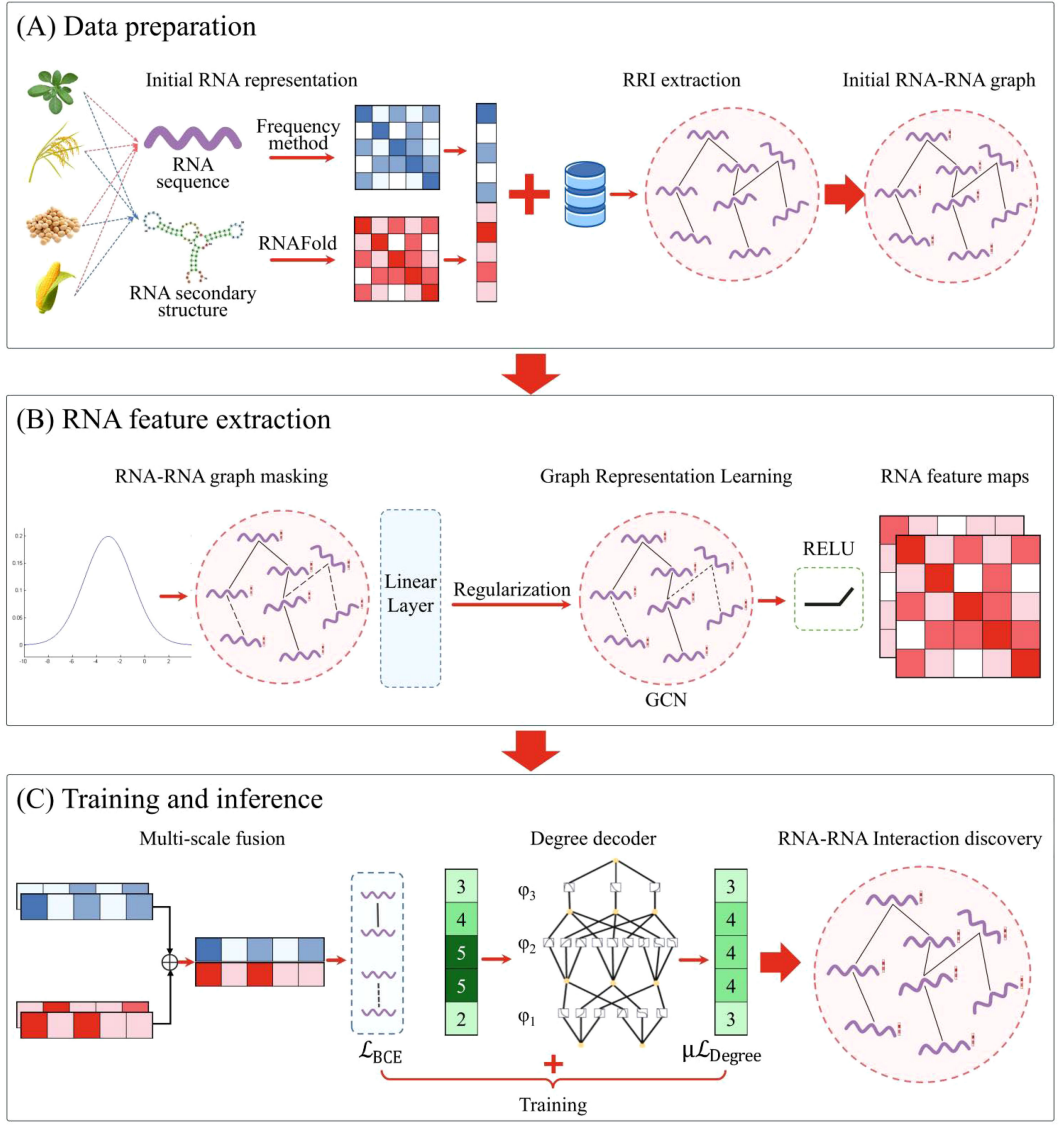
The IRGL-RRI model architecture comprises three core components: **(A)** RRI data preparation, **(B)** feature extraction through graph representation learning, and **(C)** model training and inference workflow.

As shown in **Figure 1**, the IRGL-RRI framework comprises three core modules: (A) RRI Data Preparation, (B) Graph-Based Feature Extraction, and (C) Training & Inference. Module A processes plant RNA sequences and secondary structures through k-mer frequency analysis and RNAFold-based feature extraction. Known RRIs from [Database Name] are used to construct the initial RNA-RNA graph. Module B implements Bernoulli sampling-based graph masking followed by regularized graph representation learning, producing two-layer feature embeddings. Module C integrates multi-scale features from GNN outputs through hierarchical fusion. A KAN decoder enforces biological plausibility constraints, with dual loss functions co-optimized during training. These modules operate synergistically within a unified computational framework. The masking strategy mitigates noise through stochastic node/edge masking, while graph regularization addresses message propagation biases from node degree heterogeneity. During feature extraction, these components collaboratively enhance RNA representation quality. Multi-scale fusion synthesizes hierarchical GNN outputs, preserving both local and global semantic information. The KAN decoder maintains node density consistency through nonlinear topological transformations. During RRI modeling, these components jointly boost prediction accuracy while enhancing interpretability.

#### Performing masking on RNA-RNA graph

Currently, deep learning-driven RRI prediction models focus on multimodal feature fusion (e.g., sequence conservation, secondary structure thermodynamics, spatial proximity) as well as advanced topological modeling (e.g., graphical neural networks parsing chromatin ring structure). While these approaches improve model fitting to specific datasets, they face challenges in generalization, noise robustness, and complexity. Existing techniques are often reliant on experimental databases like StarBase and LncRNASNP (Wang Q. et al., 2024), which have species bias and limited tissue coverage, hindering cross-tissue and cross-species predictions. Additionally, high-throughput experimental data from methods such as RIC-seq and CLASH (Wu T. et al., 2024) have high false-positive rates. Complex network structures, including GCN-attention mechanism fusions, are powerful but vulnerable to noise and overfitting, compromising model interpretability.

To address these challenges and building on previous work (Hou et al., 2022), this study incorporates a stochastic masking strategy into the RRI modeling process to enhance model robustness and generalization. Specifically, feature-level masking randomly masks k-mer segments or structural features (e.g., stem-loop regions) of RNA sequences using a Bernoulli distribution, prompting the model to uncover coevolutionary information among residues. Topology-level masking, on the other hand, imitates the sparsity of Hi-C data by randomly removing known RRIs from RNA-RNA graphs and combines with GAE for structural reconstruction, thereby improving adaptability to incomplete graph structures.

The RNA-RNA graph is represented as G={V,E,X} , where V denote the RNA sets, E represents the known interaction edges, and X is the initial feature matrix of the nodes. When applying the masking mechanism, we set a masking ratio α and randomly select a set R of root nodes from the graph via a Bernoulli distribution as Equation 1.


(1) R=Bernoulli(G,α)

This directs the model to focus on global structural information during training, thereby enhancing its robustness to noise and data incompleteness. The masking ratio α ranges from 0 to 1.

Using a random traversal approach, we sample paths from pre-selected root nodes to target graph structures as shown in Equation 2. During this process, known RRIs along the path are progressively removed to construct partially missing graphs. This method further enhances the model’s ability to learn under incomplete graph conditions.


(2) $$E_{mask}∼Random\;Walk\left(R,l_{walk}\right)$$

where the set of edges randomly removed from the RNA-RNA graph is denoted as $E_{mask}$ . After removing these edges, the remaining edges form the set $E-E_{mask}$ , which constitutes the masked RNA-RNA graph $G_{mask}$ . Subsequently, $G_{mask}$ is input into the IRGL-RRI model. A GCN encoder with integrated regularization is then used to learn the RNA representations.

#### Graph regularization technique

In this study, graph regularization is used to optimize the message passing process on RNA-RNA graphs, improving RNA node representation. The core idea is to apply L2 regularization before RNA feature transmission to reduce the impact of uneven node density. For the feature matrix $X={\left[x_{1},x_{2},\cdots ,x_{n}\right]}^{T}$ ,where $x_{i}$ is the feature vector of RNA ***i*** and ***n*** is the total number of RNAs, a learnable parameter matrix W is constructed to transform $x_{i}$ , producing the transformed vector $h_{i}$ , calculated as Equation 3:


(3) $$h_{i}=x_{i}W$$

Then a scaling factor c∈ℝ is introduced to regulate the number of hidden feature patterns during propagation as Equation 4:


(4) $$\vec{n_{i}}=c\frac{\vec{h_{i}}}{\left\|\vec{h_{i}}\right\|}$$

On this basis, the neural network first normalizes the transformed feature vectors to obtain the normalized representation $n_{i}$ , and further uses this to generate the final node embedding vectors as Equation 5:


(5) $$\vec{z_{i}}=\frac{1}{d_{i}+1}\vec{n_{i}}+\sum_{j\in N_{\left(i\right)}}\frac{1}{\sqrt{d_{i}+1}\sqrt{d_{j}+1}}\vec{n_{j}}$$

This process stabilizes feature propagation and enhances the model’s ability to capture structural information. Subsequently, message propagation and updating are performed on the feature matrix Z and the adjacency matrix A as Equation 6:


(6) $$GRCN\left(X,A,c\right)=c{\hat{D}}^{-\frac{1}{2}}\hat{A}{\hat{D}}^{-\frac{1}{2}}g\left(XW\right)$$

where each RNA vector is normalized using function $g\left({\left[\vec{h_{1}},\vec{h_{2}},\cdots ,\vec{h_{n}}\right]}^{T}\right)={\left[\frac{\vec{h_{1}}}{\left\|\vec{h_{1}}\right\|},\frac{\vec{h_{2}}}{\left\|\vec{h_{2}}\right\|},\cdots ,\frac{\vec{h_{n}}}{\left\|\vec{h_{n}}\right\|}\right]}^{T}$ to obtain the node embedding $Z={\left[z_{1},z_{2},\cdots ,z_{n}\right]}^{T}\in {\mathbb{R}}^{n\times f}$ . $\hat{A}=A+I_{N}$ denotes the adjacency matrix with self-loops added by incorporating the unit matrix. The degree matrix of $\hat{A}$ is represented as $\hat{D}$ .

#### Multi-scale fusion modeling of RRI

Most GNN-based models use inner products or concatenation to reconstruct RRIs after extracting features of RNA-RNA pairs. However, GNN performance depends heavily on complete neighborhood information and accurate graph structure representation. Practical strategies like Bernoulli-distribution-based edge masking in RNA-RNA graphs can lead to incomplete local structure information for some RNA nodes. This affects the accuracy of node embeddings and weakens the model’s ability to discern inter-node relationships. Traditional decoding methods also struggle to capture higher-order graph features in data-incomplete scenarios. To address these issues, this study introduces a multi-scale fusion mechanism. It combines node representations from different GNN layers through feature concatenation or inner products to compensate for performance loss due to missing structural information. However, random masking graphs as GNN inputs can introduce noise across layers, limiting fusion effectiveness and leaving a lack of mature solutions to this issue.

Drawing on previous research (Tan et al., 2023), this study employs a multi-scale fusion technique for RRI modeling. This technique uses interaction coding of RNA embedding vectors from each GNN layer and fuses them via the Hadamard product, as shown in Equation 7. This approach enhances the modeling of both local and global graph structural information, improving model robustness and representation accuracy in scenarios with incomplete information.


(7) $$q_{ij}=\|\begin{array}{c} K\\ u,v=1 \end{array}z_{i}^{\left(u\right)}⊙z_{j}^{\left(v\right)}$$

where $q_{ij}$ denotes the final representation of the RNA pair<i,j>, ⊙ represents the Hadamard product, || denotes concatenation, and K indicates the GCN layer number.

Multi-scale feature fusion critically enhances model robustness and expressive power for biological graph modeling. Recent studies demonstrate the Hadamard product’s effectiveness in amplifying local structural signals while minimizing information loss in sparse biological graphs. Compared to weighted averaging, it achieves superior sparse data modeling through signal amplification and noise suppression. Additionally, it prevents high-order semantic loss from linear fusion, and enables dynamic scale-adaptive feature weighting through subsequent mapping (Cheng et al., 2025). Biological evidence confirms that RNA functional regulation fundamentally depends on local structural interactions (base pairing, secondary structures) (Wu H. et al., 2024). This validates the biological relevance of element-wise interaction modeling (e.g., Hadamard product) for RNA interaction tasks. Our multi-scale fusion framework synergistically combines Hadamard products and concatenation to: 1) amplify local interaction signals, 2) preserve multi-granular topological semantics, and 3) optimize global feature representation for precise RRI prediction.

RNA-RNA graph modeling requires node representations that incorporate multi-scale semantic features. Lower-level features capture local topological relationships, whereas higher-level features characterize global network patterns. Given plant RNA data’s inherent structural heterogeneity and sparse interactions, our framework implements multi-scale fusion during hierarchical feature propagation. Aggregating multi-scale GNN outputs preserves local neighborhood details while incorporating global contextual information, generating comprehensive RNA representations.

#### KAN-integrated degree-aware decoder

To enhance model robustness in sparse graph structures or information masking scenarios, this study introduces a Degree-Aware Decoder integrated with the KAN technique. In RNA-RNA graphs, some nodes exhibit significant pre-/post-prediction variations. The Degree-Aware Decoder addresses this by guiding the model to better capture these discrepancies in embedded representations while improving model interpretability.

Specifically, Degree-Aware Decoder takes embeddings with topological information such as node degree as input and uses a KAN layer for nonlinear feature mapping. The KAN layer employs B-spline functions to achieve continuously differentiable local fitting and adaptively models node-degree information using trainable multi-group coefficient matrices with scale parameters. To boost model generalization, the Degree-Aware Decoder incorporates a dropout layer and an ***ELU*** activation function, enhancing nonlinear expression and robustness. This mechanism improves prediction stability under information deficiency or experimental noise and provides auxiliary signals for identifying key RNA nodes, offering better utility and biological interpretability. The Degree-Aware Decoderis defined as Equation 8:


(8) $$g_{\phi }\left(d_{i}\right)=ELU\left(Dropout\left(KAN\left(d_{v}\right)\right)\right)$$

where ϕ denotes the parameters of the degree decoder, $d_{i}$ denotes the degree of RNA ***i***, KAN(·) denotes the nonlinear transformation implemented by KANs, Dropout(·) denotes the regularization operation, and ELU(·) denotes the activation function, which introduces nonlinearity.

#### Objective function

During model training, a key loss arises from errors in modeling masked RRIs. Specifically, we randomly mask the RNA-RNA graph and input it into the GNN encoder to generate RNA molecule embeddings. The model then reconstructs the relationships between masked edges using fusion mechanisms like the cross-Hadamard product. The following objective function minimizes this RRI modeling loss, guiding the model to more accurately recover underlying RRI patterns by BCE loss as Equations 9 and 10:


(9) $${ℒ}_{BCE}=-\left({ℒ}^{+}+{ℒ}^{-}\right)$$

where


(10) $${ℒ}^{+}=\frac{1}{\left|E^{+}\right|}\sum_{\left(u,v\in E^{+}\right)}logh_{\omega }\left(z_{u},z_{v}\right),\;{ℒ}^{-}=\frac{1}{\left|E^{-}\right|}\sum_{\left(i,j\in E^{-}\right)}log\left(1-h_{\omega }\left(z_{i},z_{j}\right)\right)$$

and $z_{u}\;\text{and }z_{v}$ represent the RNA embeddings obtained by GCN encoder, $E^{+}$ denotes the set of connected RNA-RNA pairs in the graph, and $E^{-}$ represents the set of unconnected RNA-RNA pairs in the graph as Equation 11:


(11) $${ℒ}_{degree}=\frac{1}{\left|V_{M}\right|+\left|V_{D}\right|}{\sum_{i\in V_{M}\cup^{}V_{D}}\left\|f_{\phi }\left(z_{i}\right)-degree_{mask}\left(i\right)\right\|}_{F}^{2}$$

where $degree_{mask}$ denotes the set of masked nodes in the masked RNA graph and the degree of $G_{mask}$ , the overall loss is calculated as Equation 12:


(12) $$ℒ={ℒ}_{BCE}+\mu {ℒ}_{degree}$$

where μ is the adjustable parameter.

## Results

### Experimental setup

To evaluate the proposed model’s performance, we conducted comparative experiments with mainstream graph neural network models (GCN (Li et al., 2018), GAT (Veličković et al., 2018), GIN (Xu et al., 2019) and two classical miRNA-lncRNA interaction prediction methods (CIRNN (Zhang et al., 2020), LncMirNet (Yang et al., 2020)). CIRNN integrates a convolutional neural network (CNN) with an independent recurrent neural network (IRNN), showcasing strong expressive power and computational efficiency in non-coding RNA interaction prediction while supporting personalized training on private user data. To ensure fair comparison, we trained and predicted with CIRNN ten times on our study’s dataset, averaging the results as the final performance metric. Similarly, LncMirNet serves as a robust deep convolutional neural network-based prediction framework. Its core concept involves fusing four types of sequence-based information into a unified feature matrix for model input (Yang et al., 2020), and it provides pre-trained models enabling direct inference on the test set. To maintain experimental fairness, all models were evaluated on the same dataset. We utilized ten-fold cross-validation to assess the proposed model’s performance.

Additionally, the IRGL-RRI model’s initial settings included a masking rate of 0.6 and a random walk length of 0.2. Our masking strategy design accounts for RNA-RNA graphs’ inherent sparsity and noise characteristics, where random perturbations effectively simulate biological data imperfections to enhance model robustness. Probability-controlled masking (optimal masking ratio=0.6) balances information preservation and robustness enhancement. Sensitivity analysis confirmed 0.6 as the optimal masking ratio across multiple performance metrics. We therefore established 0.6 as the default masking ratio. Following previous studies (Liao et al., 2017; Ding et al., 2023; Wu et al., 2023; Cai et al., 2024; Ma et al., 2024; Qiao et al., 2024; Wang et al., 2024b; Wang et al., 2024c; Wang et al., 2024a; Wang R. et al., 2024; Wang Y. et al., 2024; Wei et al., 2024a; Wei et al., 2024b; Xie et al., 2024; Ye et al., 2024; Zhang et al., 2024; Zhao et al., 2024; Zhou et al., 2024; Feng et al., 2025; Zhou et al., 2025), we employed AUC (area under the curve), AUPR (area under the precision-recall curve), Accuracy (ACC), Precision (PRE), F1 Score (F1), and Mathews Correlation Coefficient (MCC) as evaluation metrics.

### Performance evaluation

**Figure 2:**
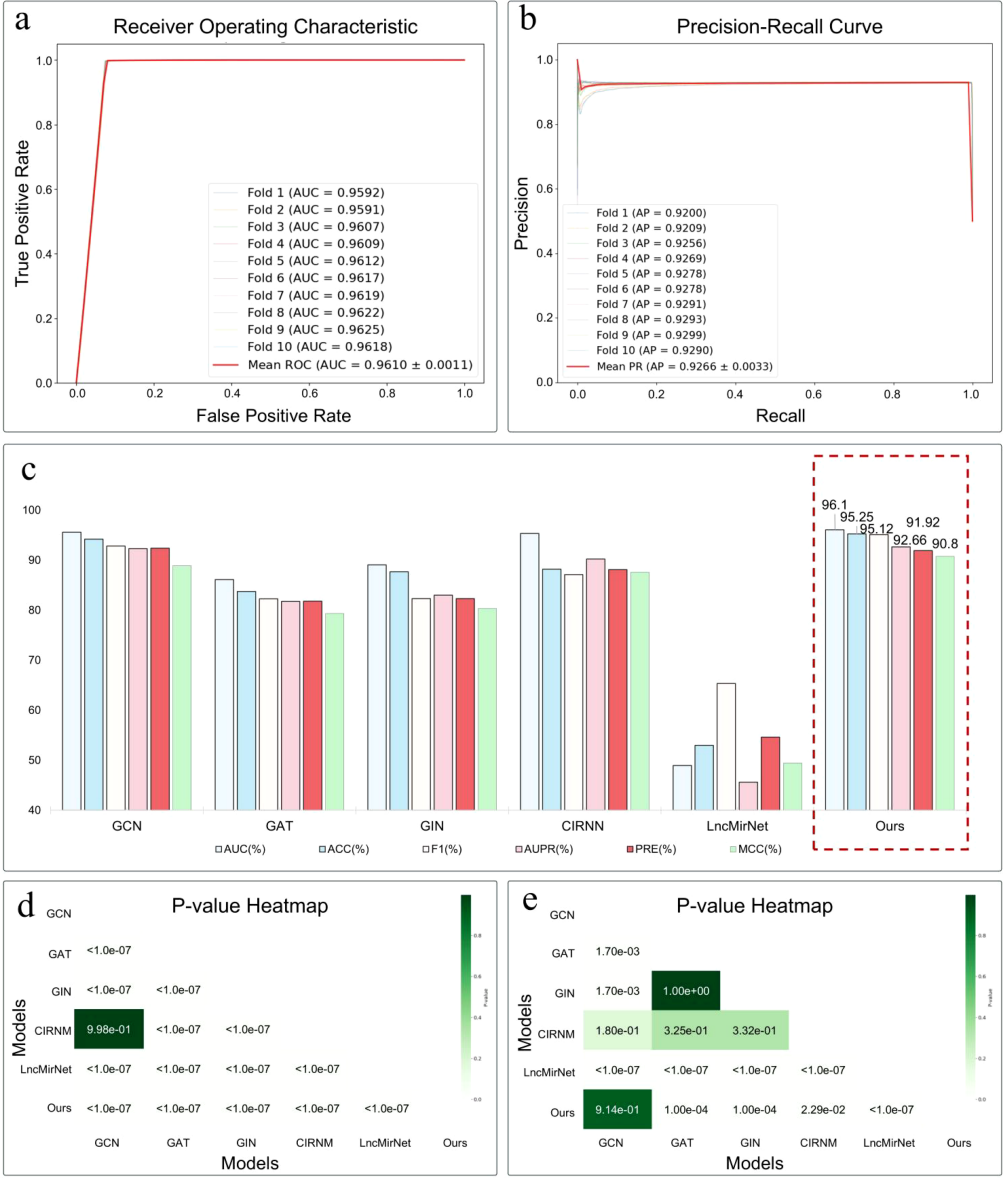
**(a)** AUC and **(b)** AUPR scores of IRGL-RRI in the ten-fold cross-validation. **(c)** Comparison of all models and Significance analysis heatmap of model performance based on **(d)** AUC and **(e)** F1 metrics.

As shown in the **Table 2** and **Figures 2a, b**, IRGL-RRI demonstrated significant advantages in ten-fold cross-validation, achieving a mean AUC of 96.10% ± 0.11. As shown in **Figure 2c**, This represents a 0.75% improvement over the current best neural network method, CIRNN (AUC 95.35%), and a 0.51% increase over the top machine learning method, GCN (AUC 95.59%). In terms of classification accuracy (ACC), our method achieved 95.25% ± 1.13, surpassing CIRNN (88.23%) and GCN (94.21%). Particularly in comprehensive discriminative ability (F1 95.12% ± 1.76), it exceeded GCN’s F1 (92.84%) by 2.28 percentage points, indicating better precision-recall balance.

**Table 2:** Results of IRGL-RRI in the ten-fold cross-validation (%).

Fold	AUC	AUPR	ACC	PRE	F1	MCC
1	95.92	92.00	92.09	88.10	90.14	84.60
2	95.91	92.09	95.38	91.91	95.50	91.12
3	96.07	92.56	95.32	91.96	95.33	90.99
4	96.09	92.69	95.46	92.31	95.52	91.13
5	96.12	92.78	95.46	92.29	95.42	91.13
6	96.17	92.78	95.76	92.55	95.88	91.82
7	96.19	92.91	95.90	92.66	96.05	92.08
8	96.22	92.93	95.81	92.54	95.96	91.89
9	96.25	92.99	95.70	92.46	95.70	91.68
10	96.18	92.90	95.62	92.44	95.68	91.56
Average	96.10 ± 0.11	92.66 ± 0.33	95.25 ± 1.13	91.92 ± 1.37	95.12 ± 1.76	90.80 ± 2.21

The stability analysis revealed that our method’s metrics have significantly lower standard deviations (e.g., AUC ± 0.11, F1 ± 1.76) than those of the comparison methods (e.g., CIRNN’s F1 standard deviation typically exceeds 3.5). This suggests that the dynamic weighting mechanism and topology-adaptive decoder used in feature construction effectively mitigate data noise interference. Through an innovative multi-scale k-mer feature fusion strategy, arithmetic progression weighting to enhance functional structural domain signals, and node-degree-based topological regularization constraints, the scale-free nature of RNA interworking networks is accurately captured. This performance breakthrough offers a more reliable computational tool for RNA interaction analysis.

### Comparison with other methods

To systematically assess the proposed model’s effectiveness, we compared its performance with various mainstream methods, including GNN approaches (GCN, GAT, GIN), deep neural network methods (CIRNN (Hou et al., 2022), LncMirNet (Tan et al., 2023)), and our proposed method. The comparison, evaluated using AUC, ACC, and F1 score, revealed that our model achieved the best performance across all three core metrics, with an AUC of 96.10%, accuracy of 95.25%, and F1 score of 95.12%, outperforming the comparison models and demonstrating strong classification ability and stability.

As shown in **Figure 2c**, among the GNN methods, the classical GCN model also performed well, achieving an AUC of 95.59% and an F1 score of 92.84%, but was slightly less accurate. In contrast, GAT and GIN showed significantly lower performance, particularly in the F1 score, suggesting they may be less sensitive to feature structures or lack sufficient generalization ability for RRI prediction. Among the deep neural network approaches, CIRNN, which combines a convolutional neural network (CNN) and an independent recurrent neural network (IRNN), has strong feature modeling capabilities and can perform personalized training on user-defined data in **Figure 2c**. After ten independent trainings and predictions using the same dataset as in this study, CIRNN achieved an average AUC of 95.35%, but its accuracy (88.23%) and F1 score (87.12%) were much lower than those of our model, indicating room for improvement in distinguishing between positive and negative samples. Another deep method, LncMirNet, performed poorly in this experiment (AUC 48.98%, ACC 52.99%), possibly due to insufficient transferability of its pre-trained model on the current dataset.

In summary, our method significantly outperforms multiple existing GNNs and deep learning models in the miRNA-lncRNA interaction prediction task. Its superior feature representation and structure modeling strategy give it a leading edge in all metrics, confirming its strong robustness and generalization ability.

### Significance analysis

This study rigorously evaluated the statistical significance of performance differences among various miRNA-lncRNA interaction prediction models using AUC and F1 metrics. A one-way ANOVA with post - hoc pairwise comparisons was applied to determine if the mean performance differences across models were statistically significant. This approach helps ascertain whether performance variations stem from the models themselves rather than chance.

Five independent experiments were conducted on six methods: GCN, GAT, GIN, CRNN, LncHNet and IRGL-RRI. The significance of AUC values for each model was analyzed, with results presented in **Figure 2d** where each cell indicates the p-value for AUC differences between two models. Most model pairs had p-values below 1.0e-07, far less than the conventional significance threshold (p< 0.05), indicating highly significant performance differences. The IRGL-RRI model showed significant performance advantages (p< 1.0e-07) over conventional methods like GCN, GAT, GIN, and LncHNet. In **Figure 2e**, for F1 metrics, most model pairs (e.g., GCN vs IGRL - RRI, GAT vs LncHNet) also had p-values below 1.0e-07, aligning with the AUC trends and further confirming IRGL- RRI’s superiority in balancing precision and recall. However, a few model pairs (e.g., CIRNN vs GCN) showed non-significant F1 differences (p =1.80e-01), likely due to feature redundancy between models.

Overall, IRGL-RRI demonstrated significant statistical advantages in both AUC and F1 analyses, particularly in handling complex RRIs. The results consistently support IRGL-RRI’s effectiveness, indicating its potential to enhance prediction performance and its high application potential in miRNA-lncRNA interaction prediction tasks.

### Parameter experiments

This section presents an in-depth assessment of model performance concerning several key adjustable hyperparameters. We focus on the weighting coefficients of auxiliary tasks in the loss function, the random masking sampling rate, and the random walk step size. By systematically varying these parameters, we analyze their effects on the model’s predictive effectiveness, thereby further verifying the model’s robustness and adaptability.

#### Performance analysis of different sampling ratios

This study delves into the effect of masking sampling rates on model performance. We incorporated a stochastic masking mechanism based on the Bernoulli distribution before inputting the miRNA-lncRNA interaction graph into the graph encoder. This mechanism generates a self-supervised learning signal by masking edges with a certain probability, simulating missing information scenarios. We set the sampling rate from 0.1 to 0.9 and fixed the random walk step size at 2, assessing model robustness under varying information loss degrees. Selecting the sampling rate requires balancing information quantity and noise suppression. A low rate may insufficiently train the model to capture interaction patterns, while a high rate may introduce redundant perturbations, damage key structural features, or cause overfitting.

**Figure 3:**
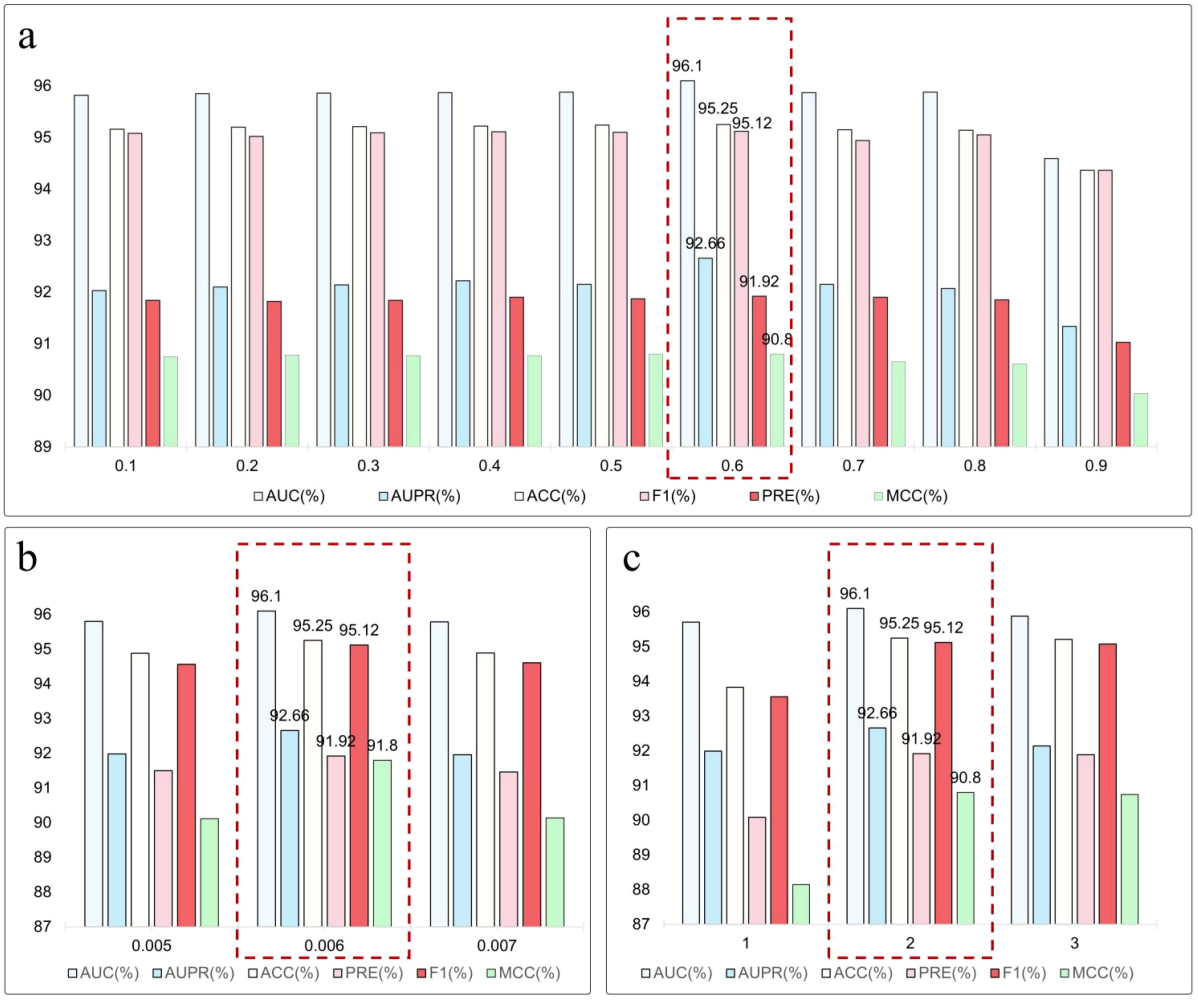
**(a)** Impact of masking ratios on model performance. **(b)** Effect of loss weighting factors ( μ ) on optimization dynamics. **(c)** Sensitivity analysis of random walk lengths in graph sampling.

Experimental results in **Figure 3a** indicate optimal model performance at a 0.6 sampling rate, achieving an AUC of 96.10%, an AUPR of 92.66%, and an F1 score of 95.12%, surpassing other rates. When the rate is below 0.3, the model’s learning ability is constrained; above 0.7, performance significantly declines, with marked regression at 0.9, where the AUC and F1 score drop to 94.59% and 94.36%, respectively. This shows excessive masking weakens graph structural integrity. In summary, a moderate random masking ratio aids in constructing effective training signals and enhances the model’s ability to generalize potential relationships in miRNA-lncRNA interaction graphs. The experiments confirm the mechanism’s effectiveness in guiding the model to learn structural information and highlight the importance of sampling rate regulation in model optimization.

#### Performance analysis of loss weight factor

To explore the auxiliary loss’s effect on the main task performance in miRNA-lncRNA interaction prediction, we set the auxiliary task weight coefficient ( μ ) at 0.005, 0.006, and 0.007. Experiments were conducted with a fixed 0.6 sampling rate and a step size of 2. Results indicate that λ = 0.006 optimizes several metrics: AUC reaches 96.10%, AUPR hits 92.66%, and F1 scores at 95.12%, as shown in **Figure 3b**. This suggests balanced synergy between primary and secondary tasks.

When μ is too low (0.005), the auxiliary task’s optimization influence is insufficient, limiting structural guidance. Conversely, when μ is too high (0.007), excessive noise may disrupt the main task’s discriminative ability. Thus, proper auxiliary task weight coefficient settings are crucial for enhancing model performance and capturing complex biological relationships.

#### Impact of different walk length

We fixed the sampling ratio at 0.6 and explored the impact of different wandering lengths on model performance, assessing their moderating effect on information coverage and structure representation during masking subgraph construction. We tested randomized wander lengths of 1, 2, and 3, and results are visualized in **Figure 3c**.

The model achieved optimal performance at a step length of 2, with an AUC of 96.10%, AUPR of 92.66%, F1 score of 95.12%, and MCC of 90.80%. At step 1, the limited masking range resulted in incomplete structural context, restricting the model’s ability to capture indirect relationships. At step 3, despite broader information coverage, performance slightly declined, likely due to noise and instability from distant neighbors. A step size of 2 effectively balances neighborhood relationship capture and noise suppression. Unlike the short path (step 1), which only captures first - order interactions, this moderate length allows comprehensive understanding of RNA molecular structural features and potential connections while avoiding redundant structural interference from longer paths. Thus, in the graph - masking - based self - supervised learning framework, random wandering step length is crucial for model learning efficiency and prediction performance. Experimental results indicate that a step length of 2 is optimal for this task.

#### Impact of different training rates

To evaluate masking efficacy across sparsity levels, we created four datasets with different training rates (10%/40%/70%/100%) representing: extreme sparsity, moderate sparsity, and complete graphs. Experimental results in **Table 3** demonstrate: 93%+ F1/AUC at 70% retention, and robust performance under extreme sparsity (10% retention: 90.63% AUC, 89.11% F1), confirming severe information loss adaptability. Notably, Bernoulli masking outperforms rule-based approaches in annotation-scarce plant ncRNA studies, requiring no prior structural/functional annotations.

**Table 3:** Results across different training rates (%).

Metrics/training rates	10%	40%	70%	100%
AUC	90.63	92.37	94.32	96.10
AUPR	86.31	89.10	90.12	92.66
ACC	89.23	91.19	92.15	95.25
F1	89.11	90.52	93.94	95.12
PRE	84.26	87.93	90.89	91.92
MCC	83.32	86.21	89.64	90.80

#### Impact of different training rates

To evaluate the Bernoulli masking strategy’s efficacy in RRI modeling, we conducted controlled experiments comparing it with the baseline model (Original) using conventional random masking. All experiments used identical datasets and training configurations. Implementation of Bernoulli masking demonstrated significant improvements across all metrics versus conventional masking. In **Table 4**, key metrics showed notable gains: F1-score (+5.56%) and Matthews correlation coefficient (+6.70%), indicating enhanced discriminative power and robustness. The mechanism achieved 96.10% AUC and 95.12% F1-score, confirming its ability to maintain representation stability under information loss or perturbations. The annotation-free masking strategy exhibits strong versatility across RNA interaction networks with varying types and sparsity levels. These results validate the masking design’s methodological value for biological graph applications.

**Table 4:** Results across different masking strategies (%).

Metrics/masking strategies	AUC	AUPR	ACC	PRE	F1	MCC
Bernoulli	96.10	92.66	95.25	91.92	95.12	90.80
Original	94.21	90.46	89.63	85.01	89.56	84.10

### Ablation study

This study systematically assessed four key components in the RRI prediction model: the path perturbation mechanism, GNN encoder with graph-structure normalization, information fusion-driven decoder, and the nonlinear mapping from KAN. Ablation and comparison experiments were designed to analyze each component’s contribution to model performance across different combinations.

For encoders, “GCN” is the standard graph convolutional network, while “L2” represents a graph encoder with L2 normalization, enhancing node representation stability and discriminative power through unit sphere mapping. For decoders, “RD” (RRI Decoder) uses Hadamard operations on miRNA-lncRNA pairs based on the GNN’s last-layer output, whereas “CD” (Collaborative Decoder) adds a multi-layer embedding fusion mechanism to capture higher-order features. In perturbation strategies, “EP” is edge-based masking via Bernoulli distribution, and “PP” uses path-based perturbation with structural reconstruction in randomly wandered subgraphs, emphasizing local path semantics. “KAN” and “MLP” represent the final output mapping modules, with KAN offering superior nonlinear modeling and feature interaction.

Results in **Table 5** show that omitting L2 normalization in the encoder or the fusion mechanism in the decoder significantly reduces model discriminative performance. This highlights normalization’s importance for stable structural feature learning and multilayer fusion’s role in boosting prediction accuracy. Path-level perturbation (PP) outperforms traditional RRI perturbation (RP) by providing more locally semantic masking signals, enhancing self-supervised learning. Notably, replacing KAN with standard MLP decreases model performance (AUC from 96.10% to 95.69%), underscoring KAN’s advantage in modeling nonlinear feature interactions and adapting to complex RNA-RNA networks.

**Table 5:** Results of ablation experiments (%).

GCN	L2	CD	RD	RP	PP	KAN	MLP	AUC	AUPR
✔			✔	✔		✔		94.52	91.26
	✔		✔		✔	✔		95.82	92.42
✔		✔			✔	✔		95.59	92.34
	✔	✔		✔		✔		95.24	92.04
	✔	✔			✔	✔		96.10	92.66
	✔	✔			✔		✔	95.69	92.12

### Case study

Gma-miRN1313, a plant-specific miRNA, holds diverse biological functions in soybean (*G. max)*. In abiotic stress response, its predicted target genes are GmNHX1 and GmP5CS, which maintain ionic homeostasis and osmoregulation, hinting at its possible role in alleviating salt and drought stresses. Similarly, Arabidopsis miR-398 enhances oxidative stress tolerance by suppressing the CSD gene, and soybean miRNAs regulate glutathione metabolism-related genes to respond to low phosphorus stress (YuLian et al., 2017). In symbiotic nitrogen fixation, Gma-miRN1313 targets GmNSP2, a key transcription factor in the nodulation signaling pathway, suggesting it may influence root nodule formation. This is akin to miR172c promoting nodulation by targeting GmNNC1. The COI1-MYC2 module’s role in jasmonate signaling-mediated symbiosis regulation indicates miRNAs can modulate symbiotic processes via hormonal signaling interactions (Liu et al., 2025). In seed lipid metabolism, Gma-miRN1313’s target genes are enriched in lipid synthesis pathways like GmDGAT1 and GmFAD2, implying it may affect fatty acid synthesis and accumulation in seeds. Likewise, miR156 regulates fatty acid metabolism by targeting SPL transcription factors, and circRNAs also regulate lipid metabolism via the miRNA axis, pointing to a conserved miRNA mechanism in metabolic regulation (Meng et al., 2024). As shown in **Table 6**, IRGL-RRI revealed 9/10 lncRNAs associated with Gma-miRN1313.

**Table 6:** Predicted lncRNA associations with Gma-miRN1313.

lncRNA	Association status	lncRNA	Association status
lcl|Gmax_Glyma.01G097300.1	Present	CNT2033862	Present
CNT2032785	Present	lcl|Gmax_Glyma.14G201400.1	Present
lcl|Gmax_Glyma.19G136600.5	Present	CNT2033385	Present
CNT2034110	Present	lcl|Gmax_Glyma.15G036500.1	Present
CNT2033264	Present	lcl|Gmax_Glyma.13G348900.4	Absent

Gma-miR169h, a soybean miR169 family member, plays crucial roles in adversity responses. The miR169 family, highly conserved in plants, regulates responses to abiotic stresses (drought, salt, low temperatures) by targeting NF-YA transcription factors (Xu et al., 2021). They affect plant acclimatization by repressing NF-YA expression. In maize (*Z. mays*), miR169 enhances salt tolerance by modulating reactive oxygen species (Xing et al., 2022). While direct studies on Gma-miR169h are lacking, it’s hypothesized to similarly aid soybean’s adversity response. In soybean, miR169c is up-regulated under drought stress, boosting transpiration but reducing drought tolerance by inhibiting GmNF-YA9. At low temperatures, miR169c down-regulation relaxes GmNFYA-C repression, activating genes like GmENOD40 to maintain rhizobial function. Under salt stress, maize miR169 enhances tolerance by regulating ROS metabolism, suggesting Gma-miR169h may also help soybeans acclimate to salt stress via a similar mechanism. As shown in **Table 7**, IRGL-RRI revealed 5/5 lncRNAs associated with Gma-miR169h.

**Table 7:** Predicted lncRNA associations with Gma-miR169h.

lncRNA	Association status	lncRNA	Association status
lcl|Gmax_Glyma.08G358000.1	Present	CNT2033407	Present
lcl|Gmax_Glyma.07G152100.1	Present	CNT2034870	Present
lcl|Gmax_Glyma.08G351600.1	Present		

Gmax_Glyma.10G164400.1 is a lncRNA located on soybean chromosome 10. It exceeds 200 nucleotides in length, lacks a significant open reading frame (ORF), and meets the classical lncRNA definition (Gao et al., 2023). The lncRNA’s promoter region and splice sites are highly conserved, suggesting it may function via RNA secondary structure or protein binding, similar to functional lncRNAs like HOTAIR in animals. Although Gmax_Glyma.10G164400.1 has low sequence conservation, evidence shows that some lncRNAs achieve functional conservation through conserved RBP sites or genomic locations (Huang et al., 2024), implying Gmax_Glyma.10G164400.1 may be regulated similarly.

Bioinformatics analysis indicates that the target genes of this lncRNA are enriched in chromatin remodeling pathways, such as SWI/SNF complex members. This suggests it may regulate gene expression by recruiting histone modifiers or epigenetic complexes. A similar mechanism was found in the soybean leaf - shape - regulatory gene Glyma.11G026400, which regulates petiole morphology via ubiquitination and gibberellin pathways (Gao et al., 2023). Gmax_Glyma.10G164400.1 shows high expression in soybean roots and significant up - regulation under salt stress. It may be involved in salt stress responses by regulating osmoregulatory genes (e.g., GmP5CS) or ion-transporting proteins (e.g., GmNHX1), aligning with the reported roles of plant lncRNAs like COOLAIR in stress responses.

In terms of tissue-specific expression, Gmax_Glyma.10G164400.1 is highly specific to floral organs. It may compete for miRNA binding through a ceRNA mechanism, regulating the stability of pollen-development-related mRNAs. This mechanism is seen in animal lncRNAs like MALAT1, which affect cell proliferation and embryonic development (Kumar et al., 2024). Studies also indicate some lncRNAs have cross-species functional conservation. For example, zebrafish and human homologs can rescue embryonic developmental defects in experiments, suggesting lncRNA function may depend on RNA structure rather than sequence. As shown in **Table 8**, IRGL-RRI revealed 5/8 miRNAs associated with Gmax_Glyma.10G164400.1.

**Table 8:** Predicted miRNA associations with Gmax_Glyma.10G164400.1.

miRNA	Association status	miRNA	Association status
gma-miR169s-5p	Present	gma-miR169d	Present
gma-miR169u	Present	Gma-miR395o	Absent
gma-miR169k	Present	Gma-miRN1289a	Absent
gma-miR169j-5p	Present	gma-miR9724	Absent

In soybean, the lncRNA CNT2032787 remains largely unstudied. However, bioinformatics analysis suggests it may play roles in epigenetic regulation, metabolic modulation, and tumor progression in plants. CNT2032787 might be located in open chromatin regions and regulate gene expression by recruiting histone modification complexes. Similarly to lncRNAs like CANT2, it may influence gene expression by regulating proto-oncogene or oncogene activity. Additionally, CNT2032787 may help tumor cells adapt to metabolic stress by modulating metabolism-related pathways and may affect cell growth and metastasis by binding to miRNAs and regulating target gene expression. It could be involved in cellular processes linked to the Wnt/β-catenin or TGF-β pathways. Clinically, CNT2032787 may serve as a liquid biopsy marker, particularly in cancers where its expression is significant, allowing non-invasive diagnosis via blood or exosomal assays. It may also be a therapeutic target, especially when combined with gene-editing techniques or epigenetic drugs. Future studies could validate CNT2032787’s functions using CRISPR technology or RNA interference and explore its diagnostic and therapeutic applications (Ding et al., 2024). As shown in **Table 9**, IRGL-RRI revealed 4/4 miRNAs associated with CNT2032787.

**Table 9:** Predicted miRNA associations with CNT2032787.

miRNA	Association status	miRNA	Association status
gma-miRN1313	Present	gma-miR169a	Present
gma-miR169s-5p	Present	gma-miR169u	Present

Before the training phase, we omitted Gma-miRN1313 and lcl|Gmax_Glyma.10G164400.1, along with their related pairwise data, from the training set. This ensured the evaluation’s independence. Using the trained model, we predicted the potential interaction between Gma-miRN1313 and lcl|Gmax_Glyma.10G164400.1. Candidate lncRNAs and miRNAs were ranked by prediction scores, and the top ten interactions were selected. Results indicated that nine miRNAs interacting with Gma-miRN1313 were confirmed in soybean-related databases. Seven of these miRNAs also showed experimentally or database-supported interactions with lcl|Gmax_Glyma.10G164400.1. These findings demonstrate the model’s strong generalization ability, even with incomplete known information. They also highlight its high accuracy and practical value in identifying novel miRNA-lncRNA interactions.

## Conclusion

This study presents an interpretable graphical representation model for accurately predicting plant RRIs. RNAs are vital for gene expression and protein synthesis, regulating spatial structures of themselves and related molecules. Yet, experimental RRI validation is costly and time-consuming. Although deep learning has enhanced prediction efficiency, existing methods still need accuracy and interpretability improvements. Here, we boost model performance through multi-scale feature extraction, a Bernoulli masking strategy, L2-regularized graph representation learning, and KAN-based multiscale fusion. Base-level features like k-mer frequency and secondary structure are extracted from RNA sequences, with higher-order feature associations reconstructed via algorithmic cascades to optimize initial RNA representations. The Bernoulli masking strategy with L2-regularized graph representation learning effectively resists noise and eases node density imbalance, enhancing node propagation. Moreover, the KAN-Integrated degree-aware decoder enables multiscale fusion of GNN layer outputs through Hadamard products and concatenation, compensating for missing information. It also models node-degree differences using B-spline functions, boosting nonlinear mapping capacity. Experimental results show our model surpasses existing methods in several metrics and proves valuable in biological applications through case studies. With interpretable graph representation learning, it promises to aid plant non-coding RNA interaction research, including molecular breeding. Future work could combine this model with CRISPR/Cas9 for result validation and extend it to cross-species interaction analysis.

The IRGL-RRI model demonstrates application potential in: (1) enhancing plant stress resilience, (2) decoding developmental regulation, and (3) optimizing molecular breeding strategies. By identifying key regulatory nodes, it provides pre-screening support for gene-editing breeding and streamlines candidate target discovery. The model’s potential in plant functional genomics includes: 1. Constructing precise RNA-RNA graphs for stress response analysis. 2. Identifying stress-specific interaction modules to optimize breeding strategies 3. Enabling cross-species RNA pattern comparisons to expand systems biology research. Additionally, future work will prioritize model-CRISPR integration through: (1) Designing sgRNA libraries targeting predicted RRI hotspots; (2) Optimizing model parameters via transfer learning using CRISPRi/a functional validation data; (3) Establishing a plant RNA interaction-phenotype database for end-to-end applications. Furthermore, the model’s interpretability module generates mechanistic hypotheses for CRISPR targeting, while experimental feedback calibrates feature weights, creating a self-iterating intelligent research paradigm.

## Data Availability

Our data and code are available at: https://github.com/Lqingquan/IGRL-RRI.
